# Association between socio-economic status and outcomes among critically ill Covid-19 adult patients in France

**DOI:** 10.1186/s13613-025-01590-5

**Published:** 2025-10-14

**Authors:** Diane Naouri, Naïke Bigé, Tai Pham, Martin Dres, Gaëtan Béduneau, Alain Combes, Antoine Kimmoun, Alain Mercat, Albert Vuagnat, Matthieu Schmidt, Alexandre Demoule, Matthieu Jamme

**Affiliations:** 1 Direction de la recherche, des études, de l’évaluation et des statistiques (DREES), Ministère du Travail, de la Santé, des Solidarités et de la Famille, Paris, France; 2 Médecine intensive - Réanimation, Institut Gustave Roussy, Villejuif, France; 3 Service de Médecine intensive – Réanimation, Hôpital du Kremlin Bicêtre, Assistance Publique Hôpitaux de Paris, Le Kremlin Bicêtre, France; 4 Service de Pneumologie et Réanimation médicale, Hôpital Pitié Salpétrière, Assistance Publique Hôpitaux de Paris, Paris, France; 5 Service de Réanimation médicale, CHU Rouen, Rouen, 76000 France; 6 Service de Médecine intensive – Réanimation, Institut de cardiologie, Assistance Publique-Hôpitaux de Paris, Hôpital Pitié-Salpêtrière, Paris, France; 7 Service de Médecine intensive – Réanimation, CHRU Nancy, Nancy, France; 8 Service de Réanimation médicale et médecine hyperbare, CHU Angers, Angers, France; 9 Service de Médecine intensive – Réanimation, Hôpital privé de l’ouest parisien, Ramsay Général de Santé, 14 rue Castiglione del Lago, Trappes, 78190 France

**Keywords:** Inequalities, Deprivation, Covid-19, Intensive care unit

## Abstract

**Introduction:**

Socio-economic inequalities have been identified as a potential risk factor for adverse outcomes in patients with Covid-19. In the specific setting of critical care, data are currently more controversial. The aim of our study is to assess the impact of social inequalities on the outcome of patients admitted to intensive care unit (ICU) for Covid-19 through a national French observational study.

**Methods:**

Based on the French administrative health care database, we identified all adults living in metropolitan France admitted in ICU for COVID-19 between March 1, 2020 and December 31, 2021. Two covariates were used to measure social vulnerability: an ecological deprivation index, the French deprivation index (Fdep), categorized in quintile (Q5 represented the most deprivated localization), and being a beneficiary of a complementary health coverage for the most deprived (CSS/AME beneficiary status). Primary outcome was in-hospital death. Secondary outcome was need for mechanical ventilation and post-acute care transfer in rehabilitation unit. Fine-Gray survival analysis or logistic regression were used according the competitive risk context. Three sensitivity analyses were performed: (1) restriction to patients admitted after January 1, 2021, adjusting for vaccination status; (2) multilevel logistic regression with a hospital-level random intercept; and (3) sex-stratified analyses.

**Results:**

There were 120 191 patients admitted to ICU with Covid-19 across metropolitan France. Among them, 29 580 (24.6%) patients lived in the most disadvantage areas and 12 462 (10.4%) were CSS/AME beneficiaries. In multivariate analysis, Fdep and CSS/AME beneficiary status were both associated with higher likelihood of in-hospital death (aSHR = 1,21 ; 95%CI = 1,16 − 1,27 for Fdep-Q5 and aSHR = 1,06 ; 95%CI = 1,01–1,11 for being beneficiary of CSS/AME) and need for invasive mechanical ventilation (aSHR = 1,16 ; 95%CI = 1,12 − 1,20 for Fdep-Q5 and aSHR = 1,06 ; 95%CI = 1,02 − 1,09 for being beneficiary of CSS/AME). Among survivors, a post-acute care transfer was negatively associated Fdep-Q5 in patients above 60 years (OR = 0.88; 95%CI = 0.81–0.94), in CSS/AME beneficiaries under 60 years (OR = 0.87; 95%CI = 0.80–0.98) as well as above 60 years (aSHR = 0.81; 95%CI = 0.74–0.88). Results were consistent across all sensitivity analyses.

**Conclusion:**

Social vulnerability was associated with higher hospital mortality, higher use of invasive mechanical ventilation and lower post-acute care transfer in rehabilitation unit in patients admitted to the ICU for COVID-19.

**Supplementary Information:**

The online version contains supplementary material available at 10.1186/s13613-025-01590-5.

## Introduction

Poor populations, who lack access to healthcare services in normal times, are the most vulnerable during crises [1]. In the context of the Covid-19 pandemic, several studies have highlighted the negative impact of social inequalities on outcomes [2, 3].

Among the hypotheses proposed to explain this association, one of the most widely supported is that socio-economic vulnerability increases exposure to the pathogen [4, 5]. It has been observed that the risk of hospital admission is higher among people living in the most densely populated areas, while the risk of hospitalization decreases with higher standards of living [6]. Moreover, once hospitalized, the impact of social inequalities appears to persist, with a higher risk of death among hospitalized Covid-19 patients living in areas with a high rate of precarious housing and overcrowded main residences [7, 8].

Beyond inequalities in infectious exposure, other explanations have been put forward for the association between social inequalities and Covid-19 prognosis. Firstly, due to its epidemic nature, hospitals in the regions most affected by Covid-19 have experienced overstretching of their capacity, resulting in an increased risk of death [9, 10]. In addition to the impact on hospital organization, it has been suggested that the most socioeconomically disadvantaged patients are more likely to have comorbidities that increase their risk of developing a severe Covid-19 [11, 12]. However, the lack of consideration of patients’ comorbidities challenges the identification of a direct causal relationship between standard of living and mortality in critically ill Covid-19 patients. Thus, although previous studies have reported an increased risk of severe disease and death in those with the lowest incomes, there is very limited evidence on the relationship between socioeconomic determinants and critical care management.

Using a large French administrative healthcare database, we sought to examine social inequalities and their association with outcomes among patients admitted to intensive care units (ICUs) for Covid-19.

## Patients and methods

### Study design and participants

Study design and participants were previously described [13]. This claims-based study used information from the French administrative health care database (Système National des Données de Santé [SNDS]). The SNDS contains data on outpatient care (medical consultation, paramedical interventions, reimbursed drug dispensation) as well as data from the French hospital discharge database (Programme de médicalisation des systèmes d’informations [PMSI]) collected during hospital stay (admission date, duration, ICD-10 codes for main and associated diagnoses, medical interventions) [14]. All these data are linked through a unique personal identification number.

We included all adult patients (> 18 years) living in mainland France (excluding overseas territories) hospitalized in French ICUs between March 1, 2020 and December 31, 2021, with a complete hospital course available and at least one ICD-10 diagnosis code for Covid-19. The complete list of ICD-10 diagnosis codes used to identify patients is provided in Appendix.

Patients with Covid-19 were classified into five groups according to the epidemic surges during which they were admitted to the ICU [5]. The first surge occurred between March 1 and June 30, 2020, the second between July 1 and December 31, 2020, the third from January 1 to June 30, 2021, the fourth between July 1 to September 30 20,121 and the fifth between October 1 and December 31. 2021.

### Variables

#### Health variables

For each ICU stay, we collected age, sex, and Simplified Acute Physiology Score (SAPS) II score [15] at ICU admission. The Charlson Comorbidity Index [16] was calculated using all ICD-10 diagnoses recorded including: myocardial infarction, chronic congestive heart failure, peripherical vascular disease, stroke and transient ischemic attack, dementia, chronic pulmonary disease, connective tissue disease, peptic ulcer disease, cirrhosis, chronic kidney disease, solid tumor, hematological malignancies and AIDS. We also collected arterial hypertension and immunocompromised status. Immunocompromised patients were defined as those with agranulocytosis, medullar aplasia, immunodeficiency, cancer treated by chemotherapy, or solid organ transplants (ICD-10 diagnosis codes are available in Appendix).

We identified oxygenation and ventilation procedures performed during hospitalization according to the French Common Classification of Medical Procedures (CCAM) [17]: invasive mechanical ventilation, non-invasive mechanical ventilation and high-flow nasal cannula (HFNC) therapy. Patients were classified according to their level of ventilation invasiveness. Mechanical ventilation was considered as the most invasive respiratory support followed by non-invasive mechanical ventilation, then HFNC therapy and finally conventional oxygenotherapy. We also collected vital status at hospital discharge as well as ICU length of stay (LOS) and hospital-LOS were calculated.

#### ICU strain

In France, all healthcare facilities are required to report their total number of beds, including those dedicated to critical care, as of December 31 each year. ICU strain was defined as the ratio of patients present in the ICU on the day of admission to the number of ICU beds declared on December 31 of the previous year.

#### Socio-economic variables

The French deprivation index (FDep) was developed to provide a neighborhood-level measure of social disadvantage specifically adapted for health studies in the French population. It reflects an accumulation of material and social disadvantages at a geographical scale and incorporate unemployment rates, proportion of blue-collar workers, proportion of population with high school diploma and median taxable households income [18]. FDep was divided into quintiles: FDep-Q1 representing the most advantaged areas and FDep-Q5 the most disadvantaged. Each patient was assigned to a FDep quintile of FDep based on their residential address.

In addition to the Fdep, we collected two further indicators of social deprivation. The first one was *Complémentaire Santé Solidaire* (CSS), a French healthcare program providing free or low-cost complementary health insurance to individuals with limited financial resources, covering costs not fully reimbursed by the national health insurance system. The second one was *Aide Médicale d’Etat* (AME), which provides healthcare access to undocumented immigrants in France who meet specific residency and income requirements, differing from CSS, which applied low-income individuals with legal residency status.

### Ethics

Condition of use and security applying to the SNDS database is defined by French government regulation dated 22 March 2017 [19]. As part of its public statistics missions, the Department for Research, Studies, Assessment, and Statistics (DREES) of the French Ministry of Health, has permanent access to the SNDS database. Public information regarding the use of the database and individual rights is available online, in accordance with the European General Data Protection Regulation n° UE 2016/679 dated 27 April 2016 [20]. In this context, and given that the study involved only anonymized administrative data with no direct patient contact, formal review by an ethics committee was not required.

### Statistical analysis

Patient characteristics were described as frequencies and percentages for categorical variables. Continuous variables were expressed as mean and standards deviation (SD) or medians and interquintile ranges (IQRs), depending on the distribution.

The association between FDep or CSS/AME beneficiary status and invasive mechanical ventilation was assessed using a competing risk framework (i.e., the Fine-Gray model), with ICU discharge alive or death in the ICU without intubation as competing events [21, 22]. The strength of the association between a specific risk factor and the event of interest in the Fine and Gray model is expressed as the sub-hazard ratio (SHR), representing the ratio of hazards associated with the cumulative incidence function in the presence and absence of the risk factor. We first estimated univariate SHR and 95% confidence intervals (CIs) for invasive mechanical ventilation. We then performed multivariate analysis adjusting for the following predefined potential confounders: age, sex, arterial hypertension, diabetes mellitus, heart disease, chronic lung disease, cirrhosis, cancer, hematological malignancies, chronic kidney disease, immunosuppression, modified SAPS II score and ICU strain. The modified SAPS II score was calculated by removing the age component, allowing age and the modified SAPS II score to be included in the same model. Models were also adjusted for the hospital’s geographic region and ICU strain to account for the potential effects of healthcare system overload. No covariate selection procedure was used, as the large number of events minimized the risk of overfitting. The proportional hazard assumption was verified using a test based on scaled Schoenfeld residuals.

Similarly, we assessed the association between the FDep or CSS/AME beneficiary status and in-hospital mortality, considering discharge alive as a competing event.

Among survivors, the association between FDep or CSS/AME beneficiary status and the probability of transfer to a post-acute care rehabilitation unit was assessed using a logistic regression model. These models were stratified by age (< 60 years and ≥60 years). Given that the availability of rehabilitation beds is unevenly distributed across France, these models were adjusted for geographic aera of residence.

We performed three sensitivity analyses. First, we repeated the main models in the subgroup of patients admitted after January 1, 2021, adjusting for vaccination status. For these patients, vaccination status was collected and classified as follows: a full vaccination scheme was defined as more than 28 days after a single dose of Ad26.COV2-S vaccine (Covid-19 Vaccine Janssen^®^) or more than 7 days after the second dose of any other vaccine; a partial vaccination was defined as less than 28 days after a single dose of Ad26.COV2-S vaccine or fewer than 7 days after the second dose and/or after the first dose of any other vaccine; patients were considered non-vaccinated if they did not receive any COVID-19 vaccine dose.

Second, we conducted a multilevel logistic regression model with a hospital-level random intercept to explicitly account for clustering of patients within hospitals, applied to in-hospital death among complete cases. The same covariates as in the main analysis were included, and the intraclass correlation coefficient (ICC) was computed to quantify the proportion of residual variance attributable to between-hospital differences.

Third, we carried out sex-stratified analyses to explore potential effect modification by sex, using the same covariates as in the main models.

A P value < 0.05 was considered significant. Analyses were computed using the SAS 2017 software (SAS Institute, Cary, NC, USA).

## Results

### Patients’ characteristics

During the study period, 120 181 patients critically ill with Covid-19 were admitted to the ICU of 668 hospitals in France. The majority (*n* = 76 612, 64%) were men and 32% (*n* = 37 962) were younger than 60 years (Table 1). A total of 12 462 patients (10.44%) were CSS/AME beneficiaries: 11 714 with CSS, 646 with AME and 102 with both. CSS/AME beneficiaries were younger than non-beneficiaries (56 +/- 14 vs. 66 +/-14 years old, p = < 0.0001). FDep score was available for 115 951 patients (96%).

**Table 1:** Characteristics of the patients admitted in ICU for Covid-19

	Fdep cohort (*n* = 115 951)					CSS/AME cohort (*n* = 120 181)	
	Q1	Q2	Q3	Q4	Q5	No	Yes
	23 479 (20,2%)	20 532 (17,7%)	21 347 (18,4%)	21 013 (18,1%)	29 580 (25,5%)	107 719 (89,6%)	12 462 (10,4%)
Age	65,8 (± 14,9)	64,8 (± 14,9)	65,9 (± 14,6)	65,8 (± 14,4)	65,5 (± 14,6)	66,4 (± 14,5)	55,8 (± 14,5)
Gender							
Men	15,279 (65,1%)	13,250 (64,5%)	13,625 (63,8%)	13,454 (64,0%)	18,296 (61,9%)	69,324 (64,4%)	7288 (58,5%)
Women	8200 (34,9%)	7282 (35,5%)	7722 (36,2%)	7559 (36,0%)	11,284 (38,2%)	38,395 (35,6%)	5174 (41,5%)
Charlson comorbidity index							
0	13,377 (57.0%)	11,376 (55,4%)	11,701 (54,8%)	11,216 (53,4%)	15,257 (51,6%)	58,593 (54,4%)	6885 (55,3%)
1–2	6950 (29,6%)	6327 (30,8%)	6642 (31,1%)	6731 (32,0%)	9910 (33,5%)	33,802 (31,4%)	3996 (32,1%)
3–4	1923 (8,2%)	1789 (8,7%)	1991 (9,3%)	2012 (9,6%)	2892 (9,8%)	9876 (9,2%)	1016 (8,2%)
5 and more	1229 (5,2%)	1040 (5,1%)	1013 (4,8%)	1054 (5,0%)	1521 (5,1%)	5448 (5,1%)	565 (4,5%)
SAPS II score	30 (21–39)	30 (20–39)	31 (22–40)	30 (21–40)	30 (20–39)	30 (21–40)	27 (18–37)
Maximal level of respiratory support							
Invasive mechanical ventilation	7412 (31,6%)	6730 (32,8%)	7281 (34,1%)	7404 (35,2%)	10,085 (34,1%)	35,814 (33,3%)	4487 (36,0%)
Non-invasive mechanical ventilation	1765 (7,5%)	1775 (8,7%)	1237 (5,8%)	1332 (6,3%)	1815 (6,1%)	7440 (6,9%)	841 (6,8%)
High flow nasal canula therapy	4134 (17,6%)	3836 (18,7%)	4495 (21,1%)	4299 (20,5%)	5312 (18,0%)	20,685 (19,2%)	2431 (19,5%)
Other oxygenotherapy	10,168 (43,3%)	8191 (39,9%)	8334 (39,0%)	7978 (38,0%)	12,368 (41,8%)	43,780 (40,6%)	4703 (37,7%)
Vasopressors use	5930 (25,3%)	5304 (25,8%)	5809 (27,2%)	5954 (28,3%)	7924 (26,8%)	28,557 (26,5%)	3389 (27,2%)
Renal replacement therapy	1633 (7,0%)	1366 (6,7%)	1386 (6,5%)	1418 (6,8%)	1964 (6,6%)	7067 (6,6%)	895 (7,2%)
ICU length of stay, days	8 (3–15)	7 (3–15)	8 (3–16)	3 (3–16)	7 (3–15)	7 (3–15)	7 (3–15)
Hospital length of stay, days	13 (8–24)	13 (7–24)	14 (8–24)	14 (8–25)	13 (7–24)	14 (8–24)	13 (8–24)
Death	5348 (22,8%)	4600 (22,4%)	5147 (24,1%)	5189 (24,7%)	7594 (25,7%)	26,475 (24,6%)	2044 (16,4%)
Surge							
First	6123 (26,1%)	4298 (20,9%)	3746 (17,6%)	3927 (18,7%)	5810 (19,6%)	21,171 (19,7%)	2758 (22,1%)
Second	5983 (25,5%)	5195 (25,3%)	5599 (26,2%)	5609 (26,7%)	7829 (26,5%)	27,083 (25,1%)	3316 (26,6%)
Third	8332 (35,5%)	7923 (38,6%)	8608 (40,3%)	8343 (39,7%)	12,114 (41,0%)	43,800 (40,7%)	4128 (33,1%)
Fourth	1442 (6,1%)	1531 (7,5%)	1605 (7,5%)	1400 (6,7%)	1837 (6,2%)	7206 (6,7%)	1328 (10,7%)
Fifth	1599 (6,8%)	1585 (7,7%)	1789 (8,4%)	1734 (8,3%)	1990 (6,7%)	8459 (7,9%)	932 (7,5%)

*Fdep* French deprivation score, *CSS* complementaire santé solidaire, *AME* aide medicale d’état, *SAPS* simplified acute physiology score, *ICU* intensive care unit

**Fig. 1:**
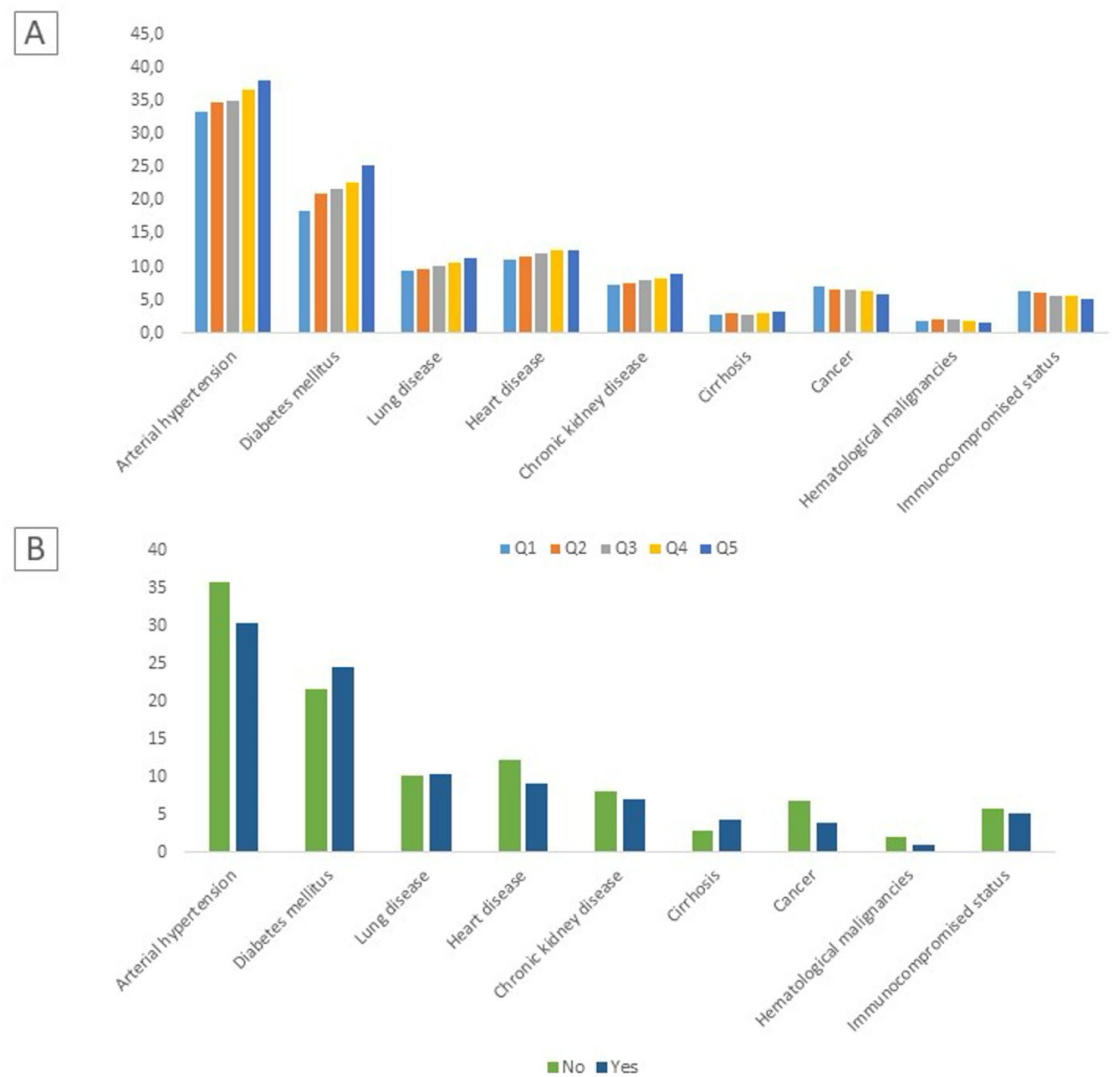
Comorbidities reported across (**A**) Fdep quintile (Q5 represented the most deprivated area) and (**B**) CSS/AME beneficiary status

Arterial hypertension (38% vs. 33%, *p* < 0.001), diabetes mellitus (25% vs. 18%, *p* < 0.001), heart disease (12% vs. 10%, *p* < 0.001), lung disease (11% vs. 9%, *p* < 0.001) and chronic kidney disease (9% vs. 7%, *p* < 0.001) were more frequent in Fdep-Q5 patients compared to Fdep-Q1 patients. In CSS/AME beneficiaries, a higher proportion had diabetes mellitus (26% vs. 22%, *p* < 0.001). Conversely, arterial hypertension (30% vs. 36%, *p* < 0.001), heart disease (9% vs. 12%, *p* < 0.001), and chronic kidney disease (7% vs. 8%, *p* < 0.001) were less frequent among CSS/AME beneficiaries (Fig. 1).

### Hospital mortality

**Fig. 2:**
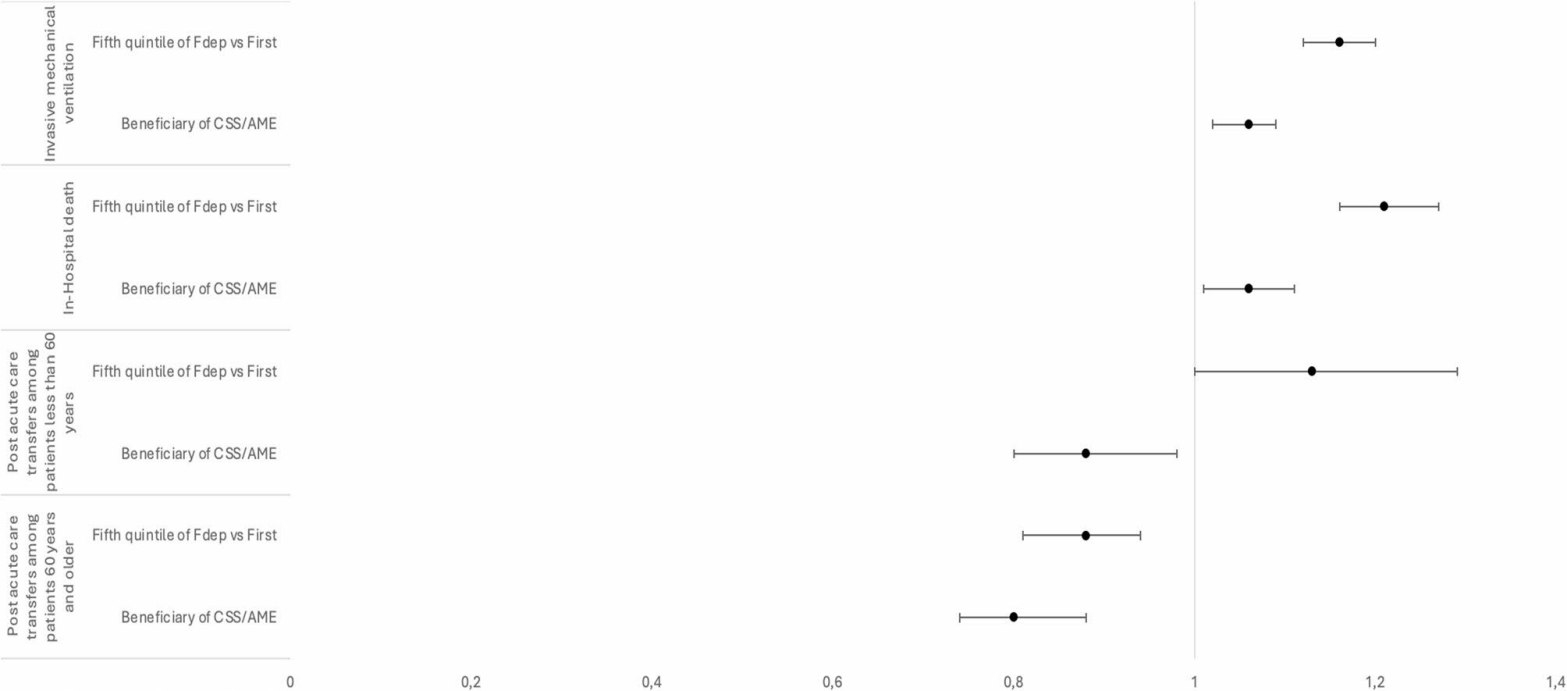
Forest plot of Fdep-Q5 and CSS/AME beneficiary status according outcomes. Point represented subdistribution hazard ratio (mechanical ventilation, in-hospital death) or odds ratio (post acute care transfert in rehabilitation unit) with the 95% confidence interval

Patients in FDep-Q5 had a higher mortality rate than those in FDep-Q1 (26% vs. 23%, *p* < 0.001). Conversely, in-hospital mortality was lower among CSS/AME beneficiaries compared with non-beneficiaries (16% vs. 25%, *p* < 0.001) (Table 1). However, in multivariate analysis, both FDep-Q5 (aSHR = 1,21 [1,16 − 1,27], *p* < 0.001) and the CSS/AME status (aSHR = 1,06 [1,01–1,11], *p* < 0.001) were statistically associated with an increased risk of in-hospital death (Fig. 2; Table 2).

**Table 2:** Fine Gray analysis of in-hospital death

	In hospital death			
	Fdep cohort (*n* = 115 951)		CSS/AME cohort (*n* = 120 181)	
	aSHR	95%CI	aSHR	95%CI
Age				
Less than 40 years	Ref		Ref	
40–49 years	1,41	1,22 − 1,64	1,47	1,28 − 1,70
50–59 years	2,41	2,12 − 2,75	2,52	2,22 − 2,86
60–69 years	4,37	3,85 − 4,96	4,62	4,09 − 5,23
70–79 years	7,83	6,89 − 8,88	8,25	7,29 − 9,33
80 years and older	20,63	18,17–23,42	21,79	19,25 − 24,67
Male gender	1,1	1,07 − 1,13	1,1	1,07 − 1,13
Comorbidities				
Arterial hypertension	0,78	0,76 − 0,80	0,78	0,76 − 0,80
Diabetes mellitus	0,96	0,93 - 0,99	0,96	0,93 - 0,99
Heart disease	1,05	1,01–1,09	1,05	1,01–1,09
Lung disease	1,08	1,04 − 1,12	1,09	1,05 − 1,13
Cirrhosis	1,36	1,28 − 1,47	1,37	1,28 − 1,47
Cancer	1,31	1,23 − 1,46	1,32	1,25 − 1,40
Hematological malignancies	0,89	0,82 − 0,98	0,89	0,82 − 0,98
Chronic kidney disease	1	0,95 − 1,04	1	0,96 − 1,05
Immunocompromised status	1,51	1,44 − 1,59	1,51	1,44 − 1,59
Modified SAPS II score				
14 or less	Ref		Ref	
15 to 20	1,41	1,35 − 1,47	1,4	1,34 − 1,46
21 to 28	1,69	1,62 − 1,77	1,68	1,61 − 1,76
29 and more	2,26	2,17 − 2,37	2,24	2,15 − 2,34
Invasive mechanical ventilation during ICU stay	1,58	1,51 − 1,66	1,6	1,53 − 1,67
RRT during ICU stay	1,77	1,71 − 1,83	1,78	1,72 − 1,84
Vasopressors during ICU stay	1,67	1,60 − 1,74	1,67	1,60 − 1,74
Surge				
First	Ref		Ref	
Second	1,06	1,03 − 1,10	1,06	1,03 − 1,10
Third	1,2	1,16 − 1,24	1,2	1,16 − 1,24
Fourth	1,2	1,12 − 1,27	1,21	1,14 − 1,28
Fifth	1,32	1,25 − 1,40	1,32	1,25 − 1,40
ICU strain				
≤ 125%	Ref		Ref	
> 125%	0,99	0,96 − 1,02	0,99	0,96 − 1,02
Quintile of FDep				
Q1	Ref			
Q2	1,07	1,02 − 1,12		
Q3	1,12	1,07 − 1,17		
Q4	1,15	1,10 − 1,20		
Q5	1,21	1,16 − 1,27		
Being a beneficiary of the CSS or AME			1,06	1,01–1,11

*Fdep* French deprivation score, *CSS* complementaire santé solidaire, *AME* aide medicale d’état, *aSHR* adjusted subdistribution hazar ratio, *SAPS* simplified acute physiology score, *RRT* renal replacement therapy, *ICU* intensive care unit

### Invasive mechanical ventilation

Invasive mechanical ventilation was more frequently used in FDep-Q5 patients (34%) compared with FDep-Q1 patients (32%, *p* < 0.001), and in CSS/AME beneficiaries compared with non-beneficiaries (36% vs. 33%, *p* < 0.001) (Table 1). In multivariate analysis, both FDep-Q5 (aSHR = 1,16 [1,12 − 1,20]) and CSS/AME beneficiary status (aSHR = 1,06 [1,02 − 1,09]) were associated with higher likelihood of requiring invasive mechanical ventilation (Fig. 2, Supplementary Table 1).

### Post acute care transfer in rehabilitation unit

Among survivors, post-acute care transfer in rehabilitation unit was more common in FDep-Q1 patients (24% vs. 23% for FDep-Q5, *p* < 0.001) and in CSS/AME beneficiaries (25% vs. 18% for non-beneficiaries, *p* < 0.001). In multivariate analysis, a negative association was observed between Fdep-Q5 (vs. FDep-Q1) and transfer to rehabilitation unit among patients aged over 60 years (OR = 0.88 [0.81–0.94]). Lower transfer rate were also found in CSS/AME beneficiaries both under 60 years (OR = 0.87 [0.80–0.98]) and over 60 years (OR = 0.81 [0.74–0.88]) (Fig. 2, Supplementary Table 2).

### Sensitive analysis

We repeated the analyses in patients admitted to ICU after January 2021, for whom vaccine status was available, with similar results (Supplementary Table 3). We did not observe difference of relationship between FDep or CSS/AME beneficiary status and in-hospital death or use of mechanical ventilation.

In the multilevel logistic regression model with a hospital-level random intercept, the associations between social vulnerability indicators (FDep-Q5 and CSS/AME beneficiary status) and in-hospital death were consistent with the main analyses. Between-hospital heterogeneity was modest, with an intraclass correlation (ICC) of 0.056 in the FDep model and 0.058 in the CMU/AME model, indicating that approximatively 6% of the residual variance in mortality risk was attributable to differences between hospitals after adjustment for covariates. The variance of the hospital random effect was 0.19 (SE 0.017) in FDep model and 0.11 (SE 0.009) in the CSS/AME model (Supplementary Table 4).

In sex-stratified analyses, a graded association between FDep quintiles and in-hospital mortality was observed in both men and women (Supplementary Fig. 1). In men with FDep-Q1 as reference, the aSHR [95% CI] were 1.07 [1.01–1.13] for FDep-Q2, 1.10 [1.05–1.16] for FDep-Q3, 1.15 [1.09–1.21] for FDep-Q4, and 1.21 [1.16–1.28] for FDep-Q5. In women with FDep-Q1 as reference, the corresponding aSHR [95% CI] were 1.07 [1.01–1.14] for FDep-Q2, 1.13 [1.06–1.20] for FDep-Q3, 1.19 [1.12–1.26] for FDep-Q4, and 1.24 [1.17–1.32] for FDep-Q5. Similarly, having CSS/AME was associated with higher in-hospital mortality in both men (aSHR = 1.08 [1.02–1.14]) and women (aSHR = 1.07 [1.01–1.13]).

## Discussion

Among all Covid-19 patients admitted to an ICU in metropolitan France during the period study, we observed an association between social vulnerability and several adverse outcomes in critically ill patients. Regardless of the measure used, higher socioeconomic disadvantage was linked to an increased risk of invasive mechanical ventilation and in-hospital death. Among survivors, CSS/AME beneficiaries had a lower likelihood of transfer to a post-acute care rehabilitation unit. Living in a deprived area was also associated with a lower likelihood of transfer, but only among patients aged over 60 years.

Since the onset of the Covid-19 pandemic, numerous studies have shown that social deprivation is associated with a higher risk of contamination [23–25], infection [23–26], hospitalization [23, 24, 27] and death [23, 26, 27]. In the specific context of critical care, some studies have suggested that the impact of deprivation persists. Using an ecological index, Lone et al. observed during the first surge (March to May 2020) that patients admitted in ICU for Covid-19 from the most deprived areas were over-represented (25%) and had an increased risk of 30-days mortality [10]. On a larger scale, and using individual-level data (migrant status, level of income, diploma) for all Swedish ICU admissions for Covid-19 requiring invasive mechanical ventilation, Nordberg et al. demonstrated an increased risk of 90-days mortality among patients of African origin and a protective effect of higher income [28].

Several hypotheses may explain the excess mortality observed in the most vulnerable patients. The first related to unequal exposure. Individuals living in the most disadvantaged areas are more likely to live in overcrowded housing, a known risk factor for SARS-CoV-2 transmission and COVID-19–related mortality [7, 8]. In our study, we also observed an association with increased use of invasive mechanical ventilation, even after adjustment for comorbidities and severity on admission. Greater exposure exposes patients to a potentially higher viral load, which has been shown to be associated with progression to the most severe forms of the disease [29]. The second hypothesis concerns a higher burden of comorbidities leading to poorer outcomes [30]. Although our results confirm an over-representation of cardiovascular and respiratory disease in the most vulnerable patients, a residual effect of social vulnerability persisted in our models after adjustment on comorbidities. However, the SNDS/PMSI database does not reliably capture several important risk factors that follow a marked social gradient in France — such as smoking status, alcohol use, and body mass index (BMI)/obesity — all of which are known to be associated with COVID-19 severity. The lack of these data represents a major limitation of our study, as residual confounding may partially explain the observed associations. The third hypothesis would be an inequality in terms of healthcare offer, in particular the density and quality of intensive care services [10]. This is especially relevant as the FDep reflects the social characteristics in population living in specific geographic areas, potentially capturing dimensions linked to local epidemic pressure and hospital resources. In our main models, adjustment for epidemic period and ICU strain accounted for part of these contextual effects. Furthermore, in our sensitivity analysis explicitly modelling hospital-level clustering, we observed modest between-hospital heterogeneity in mortality risk (ICC ≈ 6%), suggesting that hospital-level factors may may play a role, although most of the variability in outcomes was explained by individual-level factor. Nonetheless, the magnitude and direction of the associations between deprivation and mortality were not meaningfully altered, lending support to the robustness of our findings despite differences in care delivery between hospitals. The fourth hypothesis is a lower level of health literacy in the most vulnerable populations, potentially leading to lower uptake of preventive measures [31]. Our sensitivity analysis restricted to patients admitted after the start of the national vaccination campaign confirmed the protective effect of vaccination on the outcomes studied, yet the association between social deprivation and adverse outcomes persisted.

Beyond the specific context of Covid-19, assessing social inequalities and deprivation in the ICU is challenging, with studies producing contradictory results [32–38]. A recent meta-analysis reported a statistical association between social vulnerability and short-term mortality among ICU patients [39]. Interistingly, other studies - particularly from France - did not observe any difference in prognosis [37, 38, 40]. However, these studies were conducted either among homeless patients [38] or used fragility measures such as EPICES score [37], a composite index based on 11 questions related to material and social deprivation [41]. This highlights the issue of how social inequalities is measured, the heterogeneity of these measures, and theirs distinction from related concepts such as poverty or precariousness. While homelessness represents the most extreme form of precariousness, the IVOIRE study found that half the patients were classified as deprived according to EPICES score, suggesting that the score may have high sensitivity for detecting precariousness but a low positive predictive value, as also noted in another study [42]. Social inequalities refer to differences in health status observed between social groups [43]. They are not synonymous with precariousness, poverty or exclusion, but rather span all social groups along a gradient, and therefore concern society as a whole. In our study, we assessed social vulnerability using two measures: the FDep index and health coverage status. In this context, CSS/AME beneficiary status may serve as a proxy for poverty and does not capture the entire gradient between social groups. Conversely, FDep reflects socioeconomic residential conditions, offering a means of understanding social inequalities linked to the broader social environment. This raises the question of whether ecological deprivation indices can accurately reflect individual-level social inequalities – as matter still debated within the scientific community [44]. As ecological measures may underestimate the magnitude of inequalities compared to individual-level data, future studies incorporated detailed individual characteristics - such as income, educational attainment, and occupational category are warranted.

Regarding post-acute care rehabilitation transfers, we observed a negative association with social vulnerability. Limited evidence exists on social inequalities in access to post-acute care rehabilitation unit. Some studies suggest that the most vulnerable are less likely to receive rehabilitation after stroke [45] or acute coronary syndrome [46]. In the specific context of critical care, the evidence remains controversial [47]: while some studies indicate that deprivation is associated with fewer transfers to post-acute care [36], it is also established that the most vulnerable patients often experienced greater disability [48, 49] and have higher rehabilitation needs [50, 51]. However a recent French multicenter prospective study evaluating the impact of precariousness, as measured by EPICES score, on respiratory recovery in 401 Covid-19 ARDS survivors found no difference between deprived and non-deprived patients in terms of post-ICU rehabilitation [51].

Our study population included only patients admitted to the ICU for COVID-19. Therefore, our findings apply exclusively to critically ill patients and cannot be directly extrapolated to the broader population at risk of hospitalization. Assessing the influence of social vulnerability across the entire disease course — from infection to hospitalization and ICU admission — would require a different study design and data sources, and represents an important avenue for future research. Nevertheless, our results may inform targeted prevention and early access strategies for at-risk populations, support the development of equitable rehabilitation pathways, and guide benchmarking efforts to address inter-hospital variability in outcomes.

Despite the limitations outlined above, our study has several important strengths. Its nationwide scope allowed us to analyze a large and representative cohort of critically ill COVID-19 patients across all ICUs in metropolitan France. The use of two complementary indicators of socioeconomic disadvantage — one individual-level proxy (CSS/AME status) and one area-level measure (FDep index) — and the consistency of their associations across outcomes reinforce the robustness of our findings. In addition to the main analyses, we conducted multiple sensitivity analyses that strengthen the validity of our results. These included adjustment for vaccination status in the later waves of the pandemic, multilevel modelling to explicitly account for between-hospital variability, and sex-stratified analyses to explore potential effect modification. The convergence of results across these approaches supports the stability of the observed associations. Finally, our ability to adjust for ICU activity rate at the time of admission, epidemic period, and vaccination status enabled us to account for key contextual and clinical confounders that are often unavailable in large-scale administrative datasets. Taken together, these elements enhance the reliability of our findings and provide a solid basis for informing prevention strategies, promoting equitable access to post-acute care, and benchmarking inter-hospital variability in outcomes.

## Conclusion

In this nationwide study of ICU patients with COVID-19 in metropolitan France, both living in a deprived area and having CSS/AME coverage were associated with higher in-hospital mortality and lower likelihood of transfer to post-acute care rehabilitation. While consistent across several sensitivity analyses, these associations should be interpreted cautiously given residual confounding and the lack of individual-level socioeconomic data. Further research is needed to confirm these findings and guide targeted prevention and equitable care strategies.

## Supplementary Information

Supplementary material 1.

Supplementary material 2.

Supplementary material 3.

## Data Availability

The data that support the findings of this study are available from the Department for Research, Studies, Assessment, and Statistics (DREES) of the French Ministry of Health but restrictions apply to the availability of these data and so are not publicly available.
