# Supplementary material for: Association between socio-economic status and outcomes among critically ill Covid-19 adult patients in France
Source: Ann Intensive Care. 2025 Oct 14;15:159. doi: 10.1186/s13613-025-01590-5 (PMC12521692; doi:10.1186/s13613-025-01590-5)
Supplement: Supplementary file 2 — Supplementary material 2. [file 13613_2025_1590_MOESM2_ESM.docx]

**Supplementary Table 1. Multivariate Fine Gray analysis of invasive mechanical ventilation**

|  |  | **Fdep analysis** | | | **CSS/AME analysis** | | |
| --- | --- | --- | --- | --- | --- | --- | --- |
|  | | | **aSHR** | **95%CI** | | **aSHR** | **95%CI** |
| **Age** | | |  |  | |  |  |
| Less than 55 years | | | Ref |  | | Ref |  |
| 55 - 69 years | | | 1,28 | 1,24 - 1,31 | | 1,28 | 1,24 - 1,31 |
| 70 years and more | | | 0,94 | 0,91 - 0,97 | | 0,94 | 0,91 - 0,97 |
| **Male gender** | | | 1,20 | 1,17 - 1,23 | | 1,20 | 1,17 - 1,22 |
| **Comorbidities** | | |  |  | |  |  |
| Arterial hypertension | | | 1,15 | 1,13 - 1,18 | | 1,16 | 1,13 - 1,18 |
| Diabetes mellitus | | | 1,06 | 1,04 - 1,09 | | 1,07 | 1,04 - 1,09 |
| Heart disease | | | 0,96 | 0,93 - 0,99 | | 0,96 | 0,93 - 1,00 |
| Lung disease | | | 1,03 | 1,00 - 1,06 | | 1,03 | 1,00 - 1,06 |
| Cirrhosis | | | 1,00 | 0,95 - 1,06 | | 1,00 | 0,95 - 1,06 |
| Cancer | | | 0,65 | 0,61 - 0,69 | | 0,65 | 0,61 - 0,68 |
| Hematological malignancies | | | 1,27 | 1,17 - 1,39 | | 1,27 | 1,17 - 1,39 |
| Chronic kidney disease | | | 0,69 | 0,67 - 0,72 | | 0,70 | 0,67 - 0,72 |
| Immunocompromised status | | | 0,82 | 0,79 - 0,86 | | 0,83 | 0,79 - 0,87 |
| **Modified SAPS II score** | | |  |  | |  |  |
| 14 or less | | | Ref |  | | Ref |  |
| 15 to 20 | | | 2,08 | 2,00 - 2,16 | | 2,07 | 1,99 - 2,15 |
| 21 to 28 | | | 3,50 |  | | 3,49 |  |
| 29 and more | | | 6,50 | 6,27 - 6,74 | | 6,47 | 6,25 - 6,71 |
| **Surge** | | |  |  | |  |  |
| First | | | Ref |  | | Ref |  |
| Second | | | 0,63 | 0,61 - 0,65 | | 0,64 | 0,62 - 0,65 |
| Third | | | 0,66 | 0,64 - 0,67 | | 0,66 | 0,65 - 0,68 |
| Fourth | | | 0,73 | 0,70 - 0,77 | | 0,74 | 0,71 - 0,77 |
| Fifth | | | 0,56 | 0,53 - 0,59 | | 0,57 | 0,54 - 0,60 |
| **ICU strain** | | |  |  | |  |  |
| ≤ 125 % | | | Ref |  | | Ref |  |
| > 125 % | | | 1,32 | 1,29 - 1,35 | | 1,31 | 1,29 - 1,34 |
| **Quintile of FDep** | | |  |  | |  |  |
| Q1 | | | Ref |  | |  |  |
| Q2 | | | 1,08 | 1,04 - 1,12 | |  |  |
| Q3 | | | 1,10 | 1,07 - 1,14 | |  |  |
| Q4 | | | 1,13 | 1,09 - 1,17 | |  |  |
| Q5 | | | 1,16 | 1,12 - 1,20 | |  |  |
| **Being a beneficiary of the CSS or AME** | | |  |  | | 1,06 | 1,02 - 1,09 |

**Supplementary Table 2. Multivariate Logistic regression of post acute care transfert in rehabilitation unit**

|  | **Fdep Analysis** | | | | **CSS/AME analysis** | | | |
| --- | --- | --- | --- | --- | --- | --- | --- | --- |
|  | **Younger 60 years** | | **60 years and older** | | **Younger 60 years** | | **60 years and older** | |
|  | OR | 95%CI | OR | 95%CI | OR | 95%CI | OR | 95%CI |
| **Age** |  |  |  |  |  |  |  |  |
| less than 40 years | Ref |  |  |  | Ref |  |  |  |
| 40 - 49 years | 1,41 | 1,24 - 1,62 |  |  | 1,37 | 1,21 - 1,56 |  |  |
| 50 - 59 years | 1,96 | 1,73 - 2,22 |  |  | 1,90 | 1,69 - 2,14 |  |  |
| 60 - 69 years |  |  | Ref |  |  |  | Ref |  |
| 70 - 79 years |  |  | 1,69 | 1,60 - 1,77 |  |  | 1,67 | 1,59 - 1,75 |
| 80 years and older |  |  | 3,08 | 2,88 - 3,28 |  |  | 3,02 | 2,83 - 3,22 |
| **Male gender** | 0,96 | 0,89 - 1,05 | 0,83 | 0,79 - 0,87 | 0,96 | 0,88 - 1,04 | 0,84 | 0,80 - 0,88 |
| **Comorbidities** |  |  |  |  |  |  |  |  |
| Arterial hypertension | 1,08 | 0,98 - 1,18 | 1,11 | 1,06 - 1,17 | 1,09 | 0,99 - 1,19 | 1,10 | 1,05 - 1,15 |
| Diabetes mellitus | 0,95 | 0,85 - 1,05 | 1,00 | 0,94 - 1,05 | 0,96 | 0,87 - 1,06 | 1,00 | 0,95 - 1,05 |
| Heart disease | 1,26 | 1,07 - 1,49 | 1,16 | 1,09 - 1,24 | 1,26 | 1,08 - 1,48 | 1,16 | 1,09 - 1,24 |
| Lung disease | 1,15 | 1,01 - 1,31 | 1,09 | 1,02 - 1,17 | 1,17 | 1,03 - 1,33 | 1,10 | 1,02 - 1,17 |
| Cirrhosis | 1,09 | 0,89 - 1,34 | 1,02 | 0,88 - 1,17 | 1,10 | 0,91 - 1,35 | 1,02 | 0,88 - 1,17 |
| Cancer | 1,40 | 1,06 - 1,83 | 0,98 | 0,88 - 1,09 | 1,42 | 1,09 - 1,86 | 0,99 | 0,89 - 1,09 |
| Hematological malignancies | 0,74 | 0,47 - 1,16 | 1,01 | 0,84 - 1,22 | 0,70 | 0,45 - 1,08 | 0,99 | 0,83 - 1,19 |
| Chronic kidney disease | 0,93 | 0,76 - 1,15 | 0,87 | 0,80 - 0,94 | 0,90 | 0,73 - 1,10 | 0,87 | 0,80 - 0,95 |
| Immunocompromised status | 1,05 | 0,87 - 1,26 | 1,09 | 0,98 - 1,21 | 1,07 | 0,89 - 1,28 | 1,09 | 0,98 - 1,22 |
| **Modified SAPS II score** |  |  |  |  |  |  |  |  |
| 14 or less | Ref |  | Ref |  |  |  |  |  |
| 15 to 20 | 1,14 | 1,01 - 1,28 | 1,09 | 1,03 - 1,16 | 1,14 | 1,02 - 1,28 | 1,09 | 1,03 - 1,16 |
| 21 to 28 | 1,13 | 1,00 - 1,28 | 1,24 | 1,16 - 1,32 | 1,14 | 1,01 - 1,29 | 1,23 | 1,15 - 1,31 |
| 29 and more | 1,27 | 1,12 - 1,44 | 1,35 | 1,26 - 1,45 | 1,28 | 1,13 - 1,44 | 1,35 | 1,26 - 1,44 |
| **ICU length of stay, days** |  |  |  |  |  |  |  |  |
| ≤ 3 days | Ref |  | Ref |  |  |  |  |  |
| 4 - 7 days | 1,14 | 0,98 - 1,34 | 0,94 | 0,87 - 1,01 | 1,13 | 0,97 - 1,31 | 0,93 | 0,86 - 1,00 |
| 8 - 16 days | 1,95 | 1,68 - 2,26 | 1,42 | 1,32 - 1,52 | 1,93 | 1,67 - 2,23 | 1,41 | 1,32 - 1,51 |
| ≥ 17 days | 9,04 | 7,74 - 10,57 | 4,02 | 3,72 - 4,34 | 8,91 | 7,66 - 10,37 | 4,02 | 3,73 - 4,34 |
| **Invasive mechanical ventilation during ICU stay** | 3,06 | 2,76 - 3,40 | 2,62 | 2,47 - 2,78 | 3,08 | 2,78 - 3,40 | 2,62 | 2,47 - 2,77 |
| **RRT during ICU stay** | 1,39 | 1,17 - 1,64 | 1,23 | 1,10 - 1,37 | 1,39 | 1,18 - 1,65 | 1,23 | 1,10 - 1,38 |
| **Surge** |  |  |  |  |  |  |  |  |
| First | Ref |  | Ref |  |  |  |  |  |
| Second | 0,91 | 0,81 - 1,02 | 0,91 | 0,86 - 0,97 | 0,91 | 0,80 - 1,02 | 0,92 | 0,86 - 0,98 |
| Third | 1,04 | 0,94 - 1,15 | 1,10 | 1,03 - 1,17 | 1,03 | 0,93 - 1,13 | 1,08 | 1,02 - 1,15 |
| Fourth | 0,85 | 0,74 - 0,99 | 0,83 | 0,75 - 0,93 | 0,84 | 0,73 - 0,97 | 0,83 | 0,75 - 0,93 |
| Fifth | 0,43 | 0,35 - 0,53 | 0,49 | 0,44 - 0,55 | 0,42 | 0,35 - 0,51 | 0,49 | 0,44 - 0,55 |
| **Quintile of FDep** |  |  |  |  |  |  |  |  |
| Q1 | Ref |  | Ref |  |  |  |  |  |
| Q2 | 1,11 | 0,98 - 1,26 | 1,01 | 0,94 - 1,09 |  |  |  |  |
| Q3 | 1,10 | 0,96 - 1,25 | 0,99 | 0,92 - 1,07 |  |  |  |  |
| Q4 | 1,15 | 1,01 - 1,32 | 0,98 | 0,90 - 1,06 |  |  |  |  |
| Q5 | 1,13 | 1,00 - 1,29 | 0,88 | 0,81 - 0,94 |  |  |  |  |
| **Being a beneficiary of the CSS or AME** |  |  |  |  | 0,88 | 0,80 - 0,98 | 0,80 | 0,74 - 0,88 |

**Supplementary Table 3. Sensitive analysis performed on patients admitted after vaccination campaign (3rd, 4th and 5th surge)**

|  | **Invasive mechanical ventilation** | | | | **In-Hospital death** | | | |
| --- | --- | --- | --- | --- | --- | --- | --- | --- |
|  | **aSHR** | **95%CI** | **aSHR** | **95%CI** | **aSHR** | **95%CI** | **aSHR** | **95%CI** |
| **Vaccinal status** |  |  |  |  |  |  |  |  |
| Complete vaccination | 0,82 | 0,77 - 0,87 | 0,81 | 0,76 - 0,86 | 0,92 | 0,86 - 0,98 | 0,93 | 0,87 - 0,98 |
| Partial vaccination | 0,94 | 0,89 - 0,99 | 0,93 | 0,89 - 0,98 | 1,06 | 1,00 - 1,13 | 1,06 | 0,99 - 1,12 |
| No vaccination | Ref |  | Ref |  | Ref |  | Ref |  |
| **Quintile of FDep** |  |  |  |  |  |  |  |  |
| Q1 | Ref |  |  |  | Ref |  |  |  |
| Q2 | 1,10 | 1,04 - 1,15 |  |  | 1,05 | 0,97 - 1,12 |  |  |
| Q3 | 1,13 | 1,08 - 1,19 |  |  | 1,12 | 1,04 - 1,19 |  |  |
| Q4 | 1,11 | 1,06 - 1,17 |  |  | 1,17 | 1,10 - 1,24 |  |  |
| Q5 | 1,14 | 1,09 - 1,20 |  |  | 1,21 | 1,15 - 1,29 |  |  |
| **Being a beneficiary of the CSS or AME** |  |  | 1,09 | 1,04 - 1,14 |  |  | 1,12 | 1,05 - 1,20 |
| Model adjusted on age, male gender, comorbidities, SAPS II score, surge and ICU strain | | | | | | | | |

**Supplementary Table 4. Multilevel logistic regression of in-hospital death (random intercept for hospital) – associations with French deprivation index (FDep) and CSS/AME beneficiary status**

| **Variable** | **aOR** | **95% CI** | **p-value** |
| --- | --- | --- | --- |
| **Quintile of FDep** |  |  |  |
| Q1 | Ref |  |  |
| Q2 | 1.03 | 0.97 – 1.09 | 0.3128 |
| Q3 | 1.11 | 1.04 – 1.18 | 0.0012 |
| Q4 | 1.10 | 1.03 – 1.17 | 0.0014 |
| Q5 | 1.19 | 1.12 – 1.26 | <0.001 |
| **CSS/AME beneficiary** |  |  |  |
| No | Ref |  |  |
| Yes | 1.09 | 1.02 – 1.16 | 0.0103 |

**Notes:**

- aOR: adjusted odds ratio.
- Odds ratios from multilevel logistic regression models with **hospital-level random intercept** (n = 720) to account for clustering of patients within hospitals.
- Model adjusted for: age categories (<40, 40–49, 50–59, 60–69, 70–79, ≥80 years), sex, modified SAPS II quartiles (Q1–Q4, excluding age component), comorbidites (arterial hypertension, diabetes mellitus, heart disease, chronic lung disease, cirrhosis, solid cancer, hematological malignancy, chronic kidney disease, immunocompromised status), invasive mechanical ventilation, renal replacement therapy, vasopressors (catecholamines), epidemic period (1st–5th waves) & ICU activity rate (≤125% vs >125%).
- Random intercept variance: **0.1942** (FDep model) and **0.2012** (CSS/AME model), corresponding to an intraclass correlation coefficient (ICC) ≈ **0.06**.
