# Supplementary figures and images for: Association between socio-economic status and outcomes among critically ill Covid-19 adult patients in France
Source: Ann Intensive Care. 2025 Oct 14;15:159. doi: 10.1186/s13613-025-01590-5 (PMC12521692; doi:10.1186/s13613-025-01590-5)

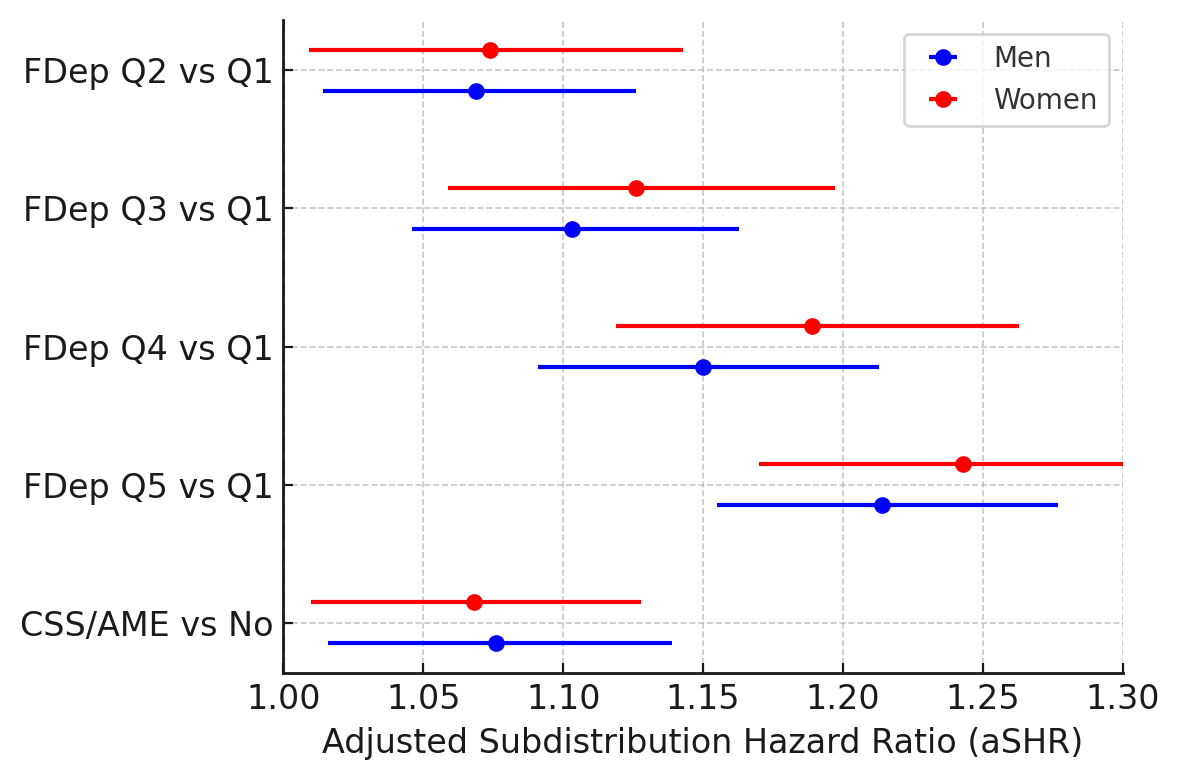

Supplement: Supplementary file 3 — Supplementary material 3. [file 13613_2025_1590_MOESM3_ESM.png]
